# Meta-analysis in a digitalized world: A step-by-step primer

**DOI:** 10.3758/s13428-024-02374-8

**Published:** 2024-04-04

**Authors:** Esther Kaufmann, Ulf-Dietrich Reips

**Affiliations:** Research Methods, Assessment, and iScience, Department of Psychology, University of Konstanz, Konstanz, Germany

**Keywords:** Meta-analysis, Overview, Research synthesis, Internet-based research, Mega-analysis, Digital research

## Abstract

In recent years, much research and many data sources have become digital. Some advantages of digital or Internet-based research, compared to traditional lab research (e.g., comprehensive data collection and storage, availability of data) are ideal for an improved meta-analyses approach.In the meantime, in meta-analyses research, different types of meta-analyses have been developed to provide research syntheses with accurate quantitative estimations. Due to its rich and unique palette of corrections, we recommend to using the Schmidt and Hunter approach for meta-analyses in a digitalized world. Our primer shows in a step-by-step fashion how to conduct a high quality meta-analysis considering digital data and highlights the most obvious pitfalls (e.g., using only a bare-bones meta-analysis, no data comparison) not only in aggregation of the data, but also in the literature search and coding procedure which are essential steps in any meta-analysis. Thus, this primer of meta-analyses is especially suited for a situation where much of future research is headed to: digital research. To map Internet-based research and to reveal any research gap, we further synthesize meta-analyses on Internet-based research (15 articles containing 24 different meta-analyses, on 745 studies, with 1,601 effect sizes), resulting in the first mega meta-analysis of the field. We found a lack of individual participant data (e.g., age and nationality). Hence, we provide a primer for high-quality meta-analyses and mega meta-analyses that applies to much of coming research and also basic hands-on knowledge to conduct or judge the quality of a meta-analyses in a digitalized world.

**Supplementary Information:**

The online version contains supplementary material available at 10.3758/s13428-024-02374-8.

## The digitalized world of internet-based research

Since its advent more than 25 years ago, Internet-based research has become a widely used research mode. In its early days, Internet-based research was considered controversial and had a difficult standing in parts of the scientific community. For example, according to Reips et al. (2016), it was argued that, because there is limited control over remote participants during the data-collection process, Internet-based research would lead to biased data. Contrary to traditional studies, in Internet-based studies more data like paradata (Heerwegh, 2003; Stieger & Reips, 2010) and drop-out data routinely become available, which could be used for further analyses and check the robustness of the results. Digitalized data are also easier to store and hence, are available for meta-analyses. Moreover, in the following years specific methods and techniques to increase the (data) quality of Internet-based science were developed. A good example is the *high hurdle technique,* which aims to exclude lowly motivated participants through an artificially levitated respondent burden (e.g., longer loading time of the first pages of a study). For an overview on additional methods and techniques, we refer to Reips (2002, 2021).

The increasing use of Internet-based research has also led to different terms for this research, e.g., “digital research” or “Internet science”. Overall, the term “Internet-based research” is a collective term for research done via the Internet, as opposed to laboratory research using computers or paper-based materials. There is a large array of terms for research conducted via the Internet; for example, the term “Internet-based experiment” is synonymous to terms such as “Web experiment, online experiment, web-based experiment, World Wide Web (WWW) experiment, and Internet experiment” (Reips, 2002, p. 243). In the following sections, the focus is on experiments as well as surveys conducted via the Internet. We consider different terms, but to avoid confusing the readers, we use “Internet-based research” throughout our primer and overview.

### Current status

Currently, Internet-based research is a widely used research approach in different fields. Scientific communities and societies have formed around meetings like the General/German Online Research Conference (GOR) that were newly founded at the time behavioral scientists discovered the Internet for conducting research or at long-standing ones like the Society for Computation in Psychology (SCiP).

Due to the resulting explosion of Internet-based research, meta-analyses within the field have already been conducted. Some advantages of Internet-based research, compared to traditional lab research (e.g., comprehensive data collection and storage, availability of data) are ideal for an improved meta-analyses approach. Although there are several meta-analyses on Internet-based research, there is no step-by-step primer available considering the pros of Internet-based research for meta-analyses and also an overview on meta-analyses within the field is missing. Such an overview shows the Internet-based research community, on what topics to best conduct the next Internet-based research studies and future meta-analyses.

Hence, in the following, we introduce meta-analysis research. We will then develop a primer for the selection of an adequate meta-analytic approach for Internet-based research, followed by a best practice example. We will then present a mega overview of research gaps to initiate follow-up meta-analyses.

## Meta-analysis

In the mid-70s, Glass (1976, 2016) introduced the term *meta-analysis*. Classical meta-analysis is known as aggregated person data meta-analysis, in which multiple studies are the analysis units. Compared to the original studies, the analysis of multiple studies has more power and reduces uncertainty. Following this, different meta-analysis approaches have been developed (Hedges & Olkin, 1985; Schmidt & Hunter, 2014; Rosenthal, 1991; for a historical overview, refer to Chalmers et al., 2002, Shadish, 2015) and therefore, without any prior knowledge on the differences between these approaches, it is unclear which approach should be used for the data aggregation. For example, in the early days, different meta-analytic approaches used the aggregation of different types of effect sizes (e.g., *d*, *r*), today the transformation of effect sizes is common (see Lipsey & Wilson, 2001).

In addition, there are two different aggregation models – the fixed effects and the random effects models. A fixed effects model assumes that all studies in the meta-analysis are derived from the same population and that the true size of an effect will be the same for all of the studies in the meta-analysis. Hence, the source of variation in the effect size is assumed to be variations within each study, such as, for instance, sampling error.

Contrary to the fixed effects model, the random effects model assumes that population effects vary from study to study. The idea behind this assumption is that the observed studies are samples drawn from a universe of studies. Random effects models have two sources of variation in a given effect size: variation arising from within studies and from variation between studies. Taking Internet-based studies as an example, we argue that the random effects model is most adequate, because Internet-based studies differ in, e.g., measurement error. Hence, we recommend to use the psychometric meta-analysis approach by Schmidt and Hunter (Hunter et al., 1982; Hunter & Schmidt, 1990; Schmidt & Hunter, 2014). In the following, we describe this approach.

### Schmidt and Hunter meta-analytic approach

Among the different ways for conducting a meta-analytic aggregation (Hedges & Olkin, 1985; Schmidt & Hunter, 2014; Rosenthal, 1991), only few follow the random effects model and consider between study differences as a source of error. Only one approach considers a palette of different sources of between study differences in detail, the so-called Schmidt and Hunter approach (Hunter et al., 1982; Hunter & Schmidt, 1990; Schmidt & Hunter, 2014). This approach leads not only to more precise estimations of the aggregated data, but also to a more precise variability estimation of data via study artifacts’ corrections (e.g., measurement error, range restriction, dichotomization; for details see Schmidt & Hunter, 2014; Kaufmann et al., 2016). Meta-analysis approaches focusing mainly on study aggregation are called *bare-bones meta-analyses*, in contrast to meta-analysis approaches that also explain the data variability caused by other study artifacts, which are called *psychometric meta-analyses* (Schmidt & Hunter, 2014). For example, it is obvious that each empirical study includes some measurement error, we never measure with 100% reliability. In addition, due to the differences in Internet-based research from lab-based research, the value of measurement error may systematically be different from traditional research. Finally, consider that without performing a psychometric meta-analysis, the between-study differences may be overestimated and “moderator variables” erroneously explaining these differences are being introduced. So there are good reasons to consider measurement error in any analyses. Therefore, we recommend theSchmidt and Hunter psychometric meta-analytic approach as the preferred meta-analytic approach for the aggregation of Internet-based research. Due to its power to correct for several between sources of variance like measurement error, the Schmidt and Hunter approach is already useful if applied to a study sample of two studies, although the small sample size needs to be discussed then (Valentine et al., 2010). Such an undertaking can provide a first estimation on between-study corrected aggregated values, which may otherwise be biased, if we considered two studies without any between-study corrections and instead made our own judgment without any statistical aggregation (see Meehl, 1954; Kaufmann & Wittmann, 2016).

Schmidt and Hunter suggested two different approaches, depending on the availability of correction data, the *individual study correction approach* or the *artifact distribution estimation approach*. Taking the correction of measurement error as an example, reliability values for each meta-analyzed study are needed for an individual study correction approach. Often such reliability values are not reported. In that case, artifact distribution estimation is the alternative strategy recommended for meta-analyses.

Independent of the chosen meta-analytic approach, for each analysis *outlier analyses* and *publication bias estimations* need to be done and critically discussed. Hence, we recommend to check the robustness of any aggregation of values by several supplemental analysis strategies.

### Advantages of meta-analyses on Internet-based research

As introduced, not using a psychometric meta-analysis is a pitfall and not using the advantages of meta-analyses on Internet-based research leads to additional pitfalls and maybe also to biased results. Hence, in the following, the we introduced two additional pitfalls to emphasize the potential of Internet-based research for meta-analyses.

#### Individual Participant Data (IPD) meta-analyses

In contrast to aggregation of studies conducted offline, Internet-based research by definition has the advantage that the data are available electronically and can easily be collected and stored without any transformation process. Hence, it is easier to retrieve data for meta-analyses (including the reliability values just discussed, and data down to the individual level), compared to research conducted offline. The recent proliferation of permanent data repositories (e.g., Vaughan, 2004) and the spread of open science policies supporting their use (see Open Science Collaboration, 2015) quickly deepened this advantage of Internet-based research.

Why is this a qualitative jump in advancing the methodology of meta-analysis? A main reason is that Individual Participant Data (IPD) meta-analyses become feasible due to the increase and easiness of collecting and storage of individual data. Instead of study-aggregated data (data that are collected by multiple individuals and combined to create a statistical report), an IPD meta-analysis considers individual participant data and prevents, therefore, aggregation bias (e.g., the *ecological fallacy*; Robinson, 1950). However, IPD meta-analyses are time- and cost-intensive with offline research, and have thus rarely been conducted. Internet-based research and the acceptance of an open science culture will substantially reduce the time and cost for conducting IPD meta-analyses (Kaufmann et al., 2016). Thus, in the future, we expect IPD meta-analyses to become a best-practice meta-analysis approach for overcoming possible aggregation bias.

#### Data comparison

A second advantage of using data collected in Internet-based research in meta-analyses is the potential to compare online data collection with offline data collection that provides a way of identifying the influence of aspects of online versus offline research modes. There is a need to consider different types of data collection approaches within meta-analyses, and we previously concluded that with its many advantages Internet-based research is a best practice approach (Kaufmann et al., 2016).

Why else do different approaches to data collection need to be considered with specific attention? For example, we refer to Hilbig and Thielmann’s (2021) critique of Thielmann et al.’s (2020) meta-analysis on deception (defined as actively providing false information to participants). They argue there is a need to carefully check the different types of data collection in terms of ethical and practical implications (e.g., whether participants in Internet-based research are not informed further when they prematurely drop out of the study) due to different circumstances. On the other hand, the degree of voluntariness (i.e., freedom to leave) throughout a study tends to be higher in online studies (Reips, 2000, 2002). Thus, in terms of ethics, Internet-based research studies run under different circumstances than, for instance, computerized lab experiments, in which participants feel socially obligated to stay and researchers can directly debrief participants who leave prematurely. Furthermore, Internet-based research involves different technical and methodological conditions that must be considered within the field as well as in data analyses (see, e.g., Honing & Reips, 2008; Kaufmann et al., 2016; Krantz & Reips, 2017; Reips, 2021).

### Summary of the state of the art

In recent years, meta-analyses became increasingly popular in several fields, such as medicine (Haidich, 2010; Menegale et al., 2023). Several guidelines were developed to evaluate meta-analyses for publication (see Preferred Reporting Items for Systematic Reviews and Meta-Analysis [PRISMA], Moher et al., 2009, Page et al., 2022; Meta-Analysis Reporting Standards [MARS], Albarracín, 2015), followed up by different primers on meta-analyses (e.g., Campos et al., 2023; Barbosa Mendes et al., 2022; Berkhout et al., 2023; Gronau et al., 2021) or specific meta-analysis topics like power-analysis (see Quintana, 2023; Valentine et al., 2010). However, up to now the advantages of Internet-based research have not been taken into account by any meta-analysis primer.

### Overviews of meta-analyses

Numerous meta-analyses can now be found in several fields; thus, it has become necessary to create overviews (*mega meta-analysis* or *review of reviews*; see, e.g., Lipsey & Wilson, 1993; for an evaluation of overviews within education, see Polanin et al., 2017) or update them due to the increase of studies in the recent years. For example, Hattie’s overview in 2009 (Hattie, 2009) included about 800 meta-analyses, his update only little more than ten years later about 2’100 meta-analyses (Hattie, 2023). Such overviews provide the scientific community and decision makers with quality checks and summaries of meta-analyses that help in finding research gaps or discerning advice for policy.^1^ A good example of an overview that includes scope and quality checks is provided by the “Mega-map on child well-being interventions in low- and middle-income countries” (UNICEF, 2022). This overview currently considers 475 systematic reviews. As it is a “living map”, it is annually updated to include new emerging evidence (i.e., new systematic reviews). Hence, the future of overviews and meta-analyses seems to be becoming more and more dynamic; this is further promoted by the evolving best practices in research that make data available for any replication or reproducible analysis (Open Science Collaboration, 2015).

From the perspective of meta-analysts, such an overview is a gain. While meta-analysis was developed to generate more precise estimations, even the most accurate meta-analysis approach is pointless if no data are available. Meta-analysts rely on original studies and need an optimal database for future re-analyses. A first meta-analytic overview of a research topic, which the present research is intended to be on Internet-based research, provides an opportunity to discover any missing information that should be reported and archived for future reuse.

### Our overall goals

To prevent readers from stepping into major pitfalls in meta-analyses (e.g., considering only a bare-bones meta-analyses, no data comparison), we provide a template, a step-by-step primer for conducting a proper meta-analysis on Internet-based research and interpreting the analyzed data correctly. Additionally, we reveal the potential of Internet-based research for meta-analyses at the current stage of meta-analysis methodology within the field, namely, with an overview of meta-analyses on Internet-based research. This follow-up overview on meta-analyses conducted within the Internet-based field also reveals topics for future studies on Internet-based research and for future meta-analysis methodology.

## A step-by-step primer on meta-analyses

In the following we will lay out the needed steps to conduct a meta-analysis. We will provide then an overview of Internet-based research and explain by example its advantages for future meta-analyses and mega meta-analyses.

Key features of each meta-analysis are *literature search*, *coding of the studies* and the *study aggregation* introduced in detail in the following (also see e.g., Siddaway et al., 2019). In our primer for meta-analyses, we add relevant information for conducting meta-analyses especially on Internet-based research. Hence, we mention how to ideally use the pros of Internet-based research (e.g., larger sample sizes) to improve the quality of the meta-analysis.

### Literature search

A comprehensive literature search is the basis of any meta-analysis. Without a carefully conducted literature search, there is a risk of only summarizing parts of the study population and running into generalization problems. A successful literature search starts with finding key articles within the field and then checks their citations (forward citation search) and references (backward citation search). Within Internet-based research key articles are e.g., Birnbaum (2004), Reips (2002, 2021), Reips et al. (2016), Skitka and Sargis (2006). Defining key articles is subjective and involves a combination of features (e.g., number of citations, written by a pioneer of the topic, or published in a high-impact journal). Because our overall goal is to find additional literature, we link the definition of key articles to publication-relevant features, which helps to find new literature.

After checking the citation of these articles for relevant literature, it should be checked whether any keywords provided in these articles or any follow-up hits include any suitable keywords for a keyword search in several databases like *PsycInfo* or *Web of Science* (see Table 1 in the Appendix). We also recommend contacting a specialist, for example a librarian at your university to support you in your search procedure. Librarians have up-to-date knowledge of which databases you have access to, about their update cycles and scope.

Each search procedure within a database needs to be documented with at least the information about the search database, the search terms, date of the literature search, the number of hits a) after the search, b) after screening the literature title and abstract and c) after reading the complete article. An ideal template to document this process is by provided by Haddaway et al. (2022, see also PRISMA flow diagram below).

In addition to a database search by keywords and citations, we also recommend to identify new studies via other search strategies. These search strategies are useful to either increase the study sample or double-check the search results and thus also are a quality check of your databased literature search. Their purpose are also to reveal nonpublished literature, gray literature (outside traditional peer-reviewed journals, see e.g., Giustini, 2019) and to prevent any publication bias. For example, web search engines (e.g., google scholar, duckduckgo.com) are helpful for conducting such a double-check. Due to the geolocation function within some web search engines, the results are dependent on the literature search location (Cooper et al., 2021; Lawal et al., 2023). Rethlefsen et al. (2021) recommend to report whether the reduction of personalization bias (e.g., using “incognito” mode in a browser) was considered in the web-based research.

A further example of such check and/or control strategies is to scrutinize programs of relevant conferences. Another recommended strategy is to contact experts within the field. Experts are identified as study authors of relevant studies or as members of relevant societies or mailing-lists. For additional literature search strategies and how to report them ideally, we refer to Rethlefsen et al. (2021).

### Coding procedure

To start the coding procedure, there is a need to formulate precise inclusion and exclusion criteria for studies, their consequences also need to be critically discussed. Because the development of a coding sheet is dynamic, we recommend to start with a couple of codes and check their availability by a pilot-coding procedure (see Brown et al., 2003; Villiger et al., 2022). Be aware that there are some codes that need extra attention.

Often only studies published in English are considered, hence, one may overlook some publications in Spanish or other languages, which leads to a language bias (Dickersin, 2005). An example of a relevant exclusion criteria is if the studies are conducted with Internet technologies, but locally within a lab or classroom, which is often the case for educational assessment tests (e.g., Programme for International Student Assessment, PISA).

 Through the comprehensive literature search and application of exclusion criteria for study selection the researcher defines the final study sample. Each of the steps we described before needs to be conducted and reported precisely. Otherwise, the generalizability of the meta-analytic results is questionable. To follow the different steps to the final study sample, the documentation with the so-called PRISMA (Preferred Reporting Items for Systematic Reviews and Meta-Analyses) flow diagram is recommended. Figure 7 gives you an example of a PRISMA flow diagram applied to our overview of meta-analyses on Internet-based research. Recently, a Shiny app was developed to foster this process (Haddaway et al., 2022). This standardized free-of charge flow diagram template has been developed to improve the reporting quality, which was criticized for many meta-analyses (see Vu-Ngoc et al., 2018). Finally, due it’s standardization, the PRISMA flow diagram provides a better reader comprehension of the review process.

**Fig. 1:**
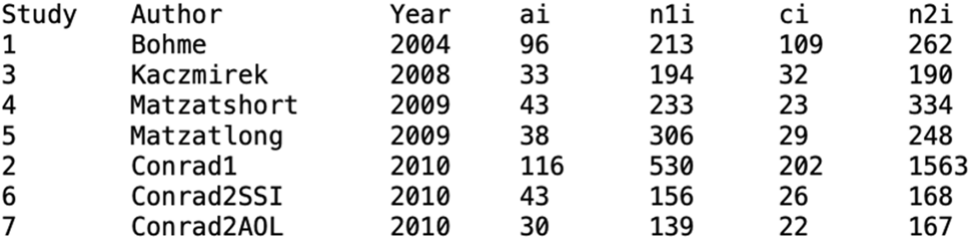
Database for Meta-Analysis Example Taken from Villar et al. (2013) (Although we use in the following the term “our database”, we emphasize that the data belong to the Villar et al. (2013) dataset, which we adapted for our analyses in R.). Note: Study = The study number; Author = The name of the study authors; Year = Publication year of the study; ai = Number of participants, who dropped off in the progress bar condition; n1i = Number of participants, who started the progress bar condition; ci = Number of participants, who dropped off in the control condition; n2i = Number of participants, who started the control condition

The overall goal of the coding procedure is to provide a comprehensive description of the studies considered and to swiftly get an overview of the study sample. The coding sheet supports the coding procedure. It includes publication (e.g., publication year) and study features, see Figure 1. Each feature needs a description and integrated quantitative code, e.g., the publication year of the study. Potential codes especially important for the evaluation of Internet-based study data quality are, e.g., if the studies reported a seriousness check, multiple site entry, warm-up or high-hurdle techniques, and if drop-out analyses are reported (for details see Reips, 2002, 2021, or below).

Internet-based research has the advantage of being able to collect large data sets from a diverse worldwide population. Thus, it needs to describe the participant sample in detail to check if this potential of Internet-based research is utilized and in what ways. Relevant sample information thus includes, in which country and which languages the study is conducted, participant age, and whether only college students were considered, to be able to assess heterogeneity and generalizability of the results.

Technical information may be important in Internet-based studies. We know that technology used may influence accessibility, timing, and results (e.g., Garaizar & Reips, 2019; Kuhlmann, Garaizar & Reips, 2021; Reips, 2021), thus it is also important to collect and aggregate data about devices used, and for example analyze whether results differ between laptop/desktop and smartphone/touchpad.

Coding features are also needed for the statistical analyses, for the aggregation of values or for the explanation of the heterogeneity by moderator variables (e.g., subgroup analyses, Borenstein et al., 2021; Schmidt & Hunter, 2014). Similar to meta-analyses on traditional studies, for meta-analyses on Internet-based research for the study aggregation, the number of participants and effect sizes for the output variables of interest need to be collected. Especially for Internet-based research is that the number of participants who dropped a survey is a useful effect size to consider for meta-analyses (see e.g., Göritz, 2006; Reips, 2002).

The coding procedure is ideally conducted by a team of experts in the field of research that is going to be meta-analyzed, they have agreed on the different codes. At least two coders are needed for any follow-up intercoder-reliability values calculations. The ReCal software by Freelon (2010; 2013) is ideal for intercoder-reliability estimation, which provides then also a quality value of the dataset for further analyses.

In Laupper et al. (2023) an online survey with a request to code the studies was sent to the first authors, this strategy saves time and increases reliability in future meta-analyses. In the same way, one should ask for initiated but not yet published projects (gray literature, see section on literature search below). We here provide the survey as a template for meta-analyses: https://ehb.eu.qualtrics.com/jfe/form/SV_agdWokJe2bMIj4y.

Text mining is a helpful supporting tool in the coding procedure of systematic reviews (Ananiadou et al., 2009; Thomas et al., 2011), as it has the potential to increase the objectivity of the review process.

Before conducting any data aggregation analyses, the description of the data should be provided first, typically summarized in a table, as shown by Shih and Fan (2008).

### Schmidt and Hunter meta-analyses

As outlined before, a psychometric meta-analysis in line with Schmidt and Hunter should be chosen for any meta-analyses. We now present a meta-analysis example step by step to introduce the reader to adequate data collection and analysis for meta-analyses on Internet-based research. For this example, we use the database from the meta-analysis by Villar et al. (2013), as this example represents a typical case. It also serves as a teaching example for courses on meta-analyses and Internet-based research.

Therefore, our step-by-step analysis and subsequent interpretation can be easily followed without any prior knowledge about meta-analysis. In our step-by-step approach, we first run a bare-bones meta-analysis, followed by the recommended psychometric meta-analysis that considers individual participant data and the difference in online vs. offline data collection.

#### Software recommendations

There are different software programs within the field. Due to the dynamic development of meta-analytic approaches, we recommend *R* (R Core Team, 2021) and specifically the *Psychmeta* (Dahlke & Wiernik, 2018) and *metafor* (Viechtbauer, 2010) packages to conduct the suggested Schmidt and Hunter (2014) analyses. An additional program, which is less flexible because it integrates not all R-packages, but is ideal for the first steps in meta-analysis and is also helpful for adaptations of figures (e.g., forest plots) is *JASP* (JASP Team, 2023). To run our example step-by-step analyses, we here use the R-program *metafor* by Viechtbauer (2010).

#### Preparation: database and upload to R

For our step-by-step analyses, we take a subsample of the meta-analysis by Villar et al. (2013), the seven studies classified within the label: slow-to-fast (see Figure 1). You will also find the documentation for the analyses on the submission platform of this manuscript (see Example_Primer2023.txt).

This meta-analysis considers typical Internet-based research as they focus on progress indicators for web surveys.

Our database is prepared as a .txt document to be easily used for analysis in R and saved as Slow_AutorYear.

After opening the program R and installing the metafor packages, we first open our data set in R with the following R command (R commands are bold in the following):

$$\mathbf d\mathbf a\mathbf t<-\mathbf r\mathbf e\mathbf a\mathbf d.\mathbf t\mathbf a\mathbf b\mathbf l\mathbf e(\text{"}\mathbf S\mathbf l\mathbf o\mathbf w\_\mathbf A\mathbf u\mathbf t\mathbf o\mathbf r\mathbf Y\mathbf e\mathbf a\mathbf r.\mathbf t\mathbf x\mathbf t\text{"},\mathbf h\mathbf e\mathbf a\mathbf d\mathbf e\mathbf r=\mathbf T)$$

Our text document (txt.data file) is linked with R and assigned to the object: dat. Here you can also use a different name than “dat”. Make sure you enter the correct NAME of your data file, in our example: Slow_AutorYear. Header = T: Header, our title is included so that we know and can check the column names.

$$\mathbf{a}\mathbf{t}\mathbf{t}\mathbf{a}\mathbf{c}\mathbf{h}(\mathbf{d}\mathbf{a}\mathbf{t})$$

Is used to link your data file.

#### Bare-Bones Meta-Analysis

First, we perform a bare-bones meta-analysis. We start it with the calculation of Odds Ratio values for the available data with the following command:

$$\mathbf d\mathbf a\mathbf t<-\mathbf e\mathbf s\mathbf c\mathbf a\mathbf l\mathbf c(\mathbf m\mathbf e\mathbf a\mathbf s\mathbf u\mathbf r\mathbf e=\text{"}\mathbf{OR}\text{"},\mathbf a\mathbf i=\mathbf a\mathbf i,\mathbf n1\mathbf i=\mathbf n1\mathbf i,\mathbf c\mathbf i=\mathbf c\mathbf i,\mathbf n2\mathbf i=\mathbf n2\mathbf i,\mathbf d\mathbf a\mathbf t\mathbf a=\mathbf d\mathbf a\mathbf t)$$

Please note that escalc is a transformation command. With the expression “measure” we specify what the target measure should be. In our case it is Odds Ratio, hence “OR”. R then specifies values that must be associated with the data set (ai, n1i, ci, n2i). We have already prepared our data set in such a way that we always have the same designations as R specifies, therefore ai=ai, n1i=n1i, etc. With the command data, we assign our dataset (“dat”) again.

**Fig. 2:**
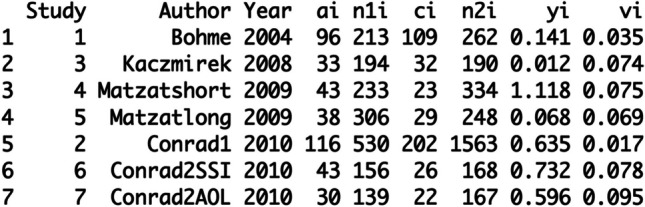
Estimating the Odds Ratio in our Database

Before we start, we prepare our database, because for each study we’ll need to calculate the appropriate correlation value. Our current database is visible with the print command (see Figure 2):

“yi” represents the newly calculated Odds Ratio values for each study and “vi” is the weighting value. Historically, the Schmidt and Hunter meta-analytic approach used as a weighing strategy the number of participants in each study (e.g., Hunter & Schmidt, 1990). Today, meta-analytic studies are often weighing by the inverse of the variance. The inverse of variance considers the precision of the estimation, not only the number of participants. In the current example, we used the inverse weighing strategy, but also recommend to compare different weighing strategies to check for the robustness of the results.

To transfer Odds Ratio to correlation values, use the following command, in the example below for the first Odds Ratio value, and add the resulting value to your database. Repeat for each Odds Ratio value.

$$\begin{array}{l}\mathbf{l}\mathbf{i}\mathbf{b}\mathbf{r}\mathbf{a}\mathbf{r}\mathbf{y}(\mathbf{e}\mathbf{f}\mathbf{f}\mathbf{e}\mathbf{c}\mathbf{t}\mathbf{s}\mathbf{i}\mathbf{z}\mathbf{e})\\ \mathbf{o}\mathbf{d}\mathbf{d}\mathbf{s}\mathbf{r}\mathbf{a}\mathbf{t}\mathbf{i}\mathbf{o}\_\mathbf{t}\mathbf{o}\_\mathbf{r}(0.1413)\end{array}$$

**Fig. 3:**
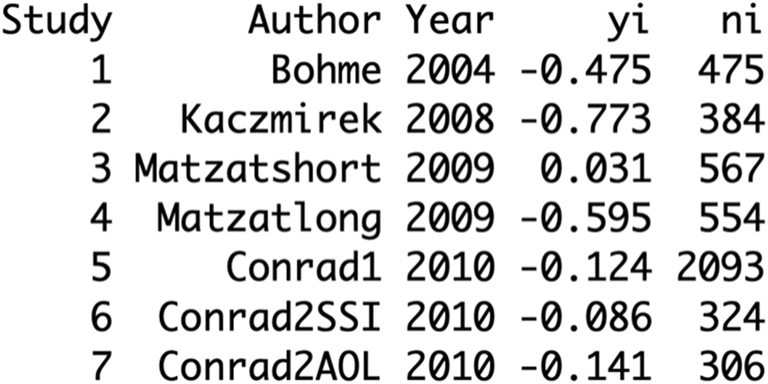
Change the Odds Ratio to Correlation Values and Add the Total Sample Size to the Database

The command above results in the following correlation value: -0.475. The new dataset includes now the “yi” value new as a correlation. In addition, you then write the total sample size of each study to the database txt.file and label it “ni”. Hence, you get the following database (see Fig. 3):

Now, you need to change the dataset as follows:

$$\mathbf d\mathbf a\mathbf t<-\mathbf e\mathbf s\mathbf c\mathbf a\mathbf l\mathbf c(\mathbf m\mathbf e\mathbf a\mathbf s\mathbf u\mathbf r\mathbf e=\text{"}\mathbf{COR}\text{"},\mathbf r\mathbf i=\mathbf y\mathbf i,\mathbf n\mathbf i=\mathbf n\mathbf i,\mathbf d\mathbf a\mathbf t\mathbf a=\mathbf d\mathbf a\mathbf t)$$

With this command you link the “yi” variable with the term “ri” (correlation) and “ni” with the total number of sample sizes (“ni”) and calculate the “vi” = sample variance.

**Fig. 4:**
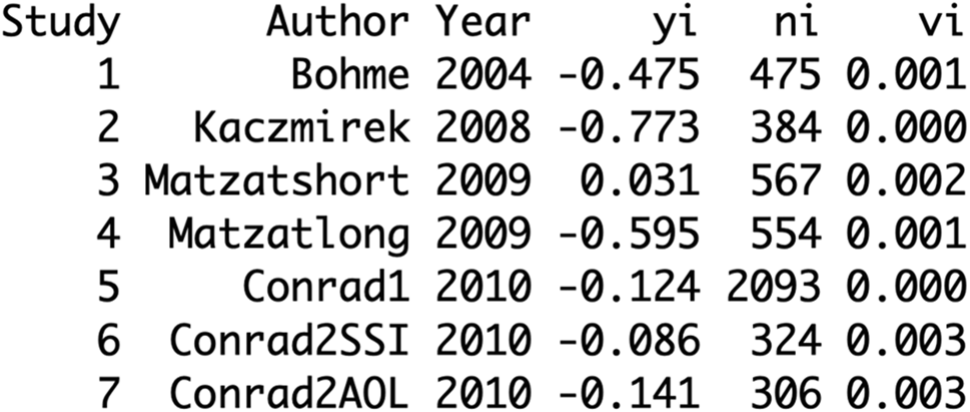
The Database with the Added Column Sample Variance

The column “vi” is added to your database (see Fig. 4):

With the following command you run a first meta-analysis:

$$\begin{array}{l}\mathbf r\mathbf e\mathbf s<-\mathbf r\mathbf m\mathbf a(\mathbf y\mathbf i,\mathbf v\mathbf i,\mathbf w\mathbf e\mathbf i\mathbf g\mathbf h\mathbf t\mathbf s=1/\mathbf v\mathbf i,\mathbf d\mathbf a\mathbf t\mathbf a=\mathbf d\mathbf a\mathbf t,\mathbf m\mathbf e\mathbf t\mathbf h\mathbf o\mathbf d=\text{"}\mathbf{HS}\text{"})\\\mathbf r\mathbf e\mathbf s\end{array}$$

**Fig. 5:**
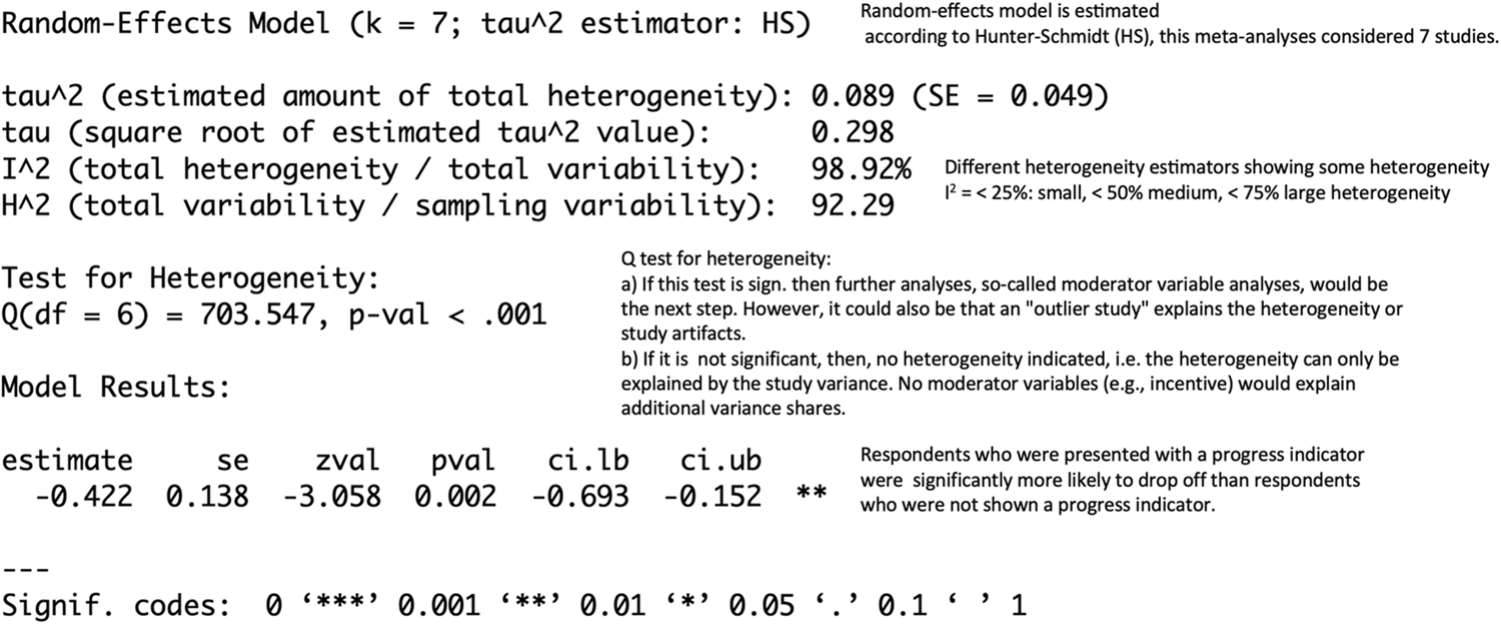
Commented Output of your Bare-Bones Meta-Analysis

This results in the following output (see Figure 5):

To summarize, our results confirmed Villar's analyses and showed that respondents who were presented with a progress indicator that showed slow progress at the beginning of the survey and then sped up were more likely to drop off than respondents who were not shown a progress indicator.

As mentioned, there is heterogeneity within the database, which needs to be explored. Therefore, an outlier analysis and a publication bias estimation are indicated to check the robustness of the results. However, as outlined before, within the Schmidt and Hunter approach, heterogeneity may also be a reason for study artifacts. Therefore, we show in the following an example of how to correct it.

#### Psychometric Corrections and Meta-Analytical Comparison

Unfortunately, our database does not contain any reliability values. We illustrate the analysis by generating some reasonable reliability values to check the robustness of our previously suggested meta-analytic heterogeneity results. Therefore, we assume reliability values in line with the *artifact distribution approach* suggested by Hunter and Schmidt. We consider reliability values from 0.95 to 0.99 for both the control and the experimental conditions. Please note that these reliability values are quite conservative.

With the following command lines, you can construct an artifact distribution and use it to correct the correlation values (the last two lines). The set command makes the estimation replicable.

$$\begin{array}{l}\mathbf{s}\mathbf{e}\mathbf{t}.\mathbf{s}\mathbf{e}\mathbf{e}\mathbf{d}(513131)\\ \mathbf{d}\mathbf{a}\mathbf{t}\mathbf{\$}\mathbf{r}\mathbf{e}\mathbf{l}\mathbf{x}<-\mathbf{r}\mathbf{o}\mathbf{u}\mathbf{n}\mathbf{d}(\mathbf{r}\mathbf{u}\mathbf{n}\mathbf{i}\mathbf{f}(\mathbf{n}\mathbf{r}\mathbf{o}\mathbf{w}(\mathbf{d}\mathbf{a}\mathbf{t}),0.95,0.99),2)\\ \begin{array}{l}\mathbf{d}\mathbf{a}\mathbf{t}\mathbf{\$}\mathbf{r}\mathbf{e}\mathbf{l}\mathbf{y}<-\mathbf{r}\mathbf{o}\mathbf{u}\mathbf{n}\mathbf{d}(\mathbf{r}\mathbf{u}\mathbf{n}\mathbf{i}\mathbf{f}(\mathbf{n}\mathbf{r}\mathbf{o}\mathbf{w}(\mathbf{d}\mathbf{a}\mathbf{t}),0.95,0.99),2)\\ \mathbf{d}\mathbf{a}\mathbf{t}\mathbf{\$}\mathbf{y}\mathbf{i}.\mathbf{c}<-\mathbf{d}\mathbf{a}\mathbf{t}\mathbf{\$}\mathbf{y}\mathbf{i}/\mathbf{s}\mathbf{q}\mathbf{r}\mathbf{t}(\mathbf{d}\mathbf{a}\mathbf{t}\mathbf{\$}\mathbf{r}\mathbf{e}\mathbf{l}\mathbf{x}\mathbf{*}\mathbf{d}\mathbf{a}\mathbf{t}\mathbf{\$}\mathbf{r}\mathbf{e}\mathbf{l}\mathbf{y})\\ \mathbf{d}\mathbf{a}\mathbf{t}\mathbf{\$}\mathbf{v}\mathbf{i}.\mathbf{c}<-\mathbf{d}\mathbf{a}\mathbf{t}\mathbf{\$}\mathbf{v}\mathbf{i}/(\mathbf{d}\mathbf{a}\mathbf{t}\mathbf{\$}\mathbf{r}\mathbf{e}\mathbf{l}\mathbf{x}\mathbf{*}\mathbf{d}\mathbf{a}\mathbf{t}\mathbf{\$}\mathbf{r}\mathbf{e}\mathbf{l}\mathbf{y})\end{array}\end{array}$$

You then rerun your meta-analyses with the following command:

$$\begin{array}{l}\mathbf r\mathbf e\mathbf s<-\mathbf r\mathbf m\mathbf a(\mathbf y\mathbf i.\mathbf c,\mathbf v\mathbf i.\mathbf c,\mathbf w\mathbf e\mathbf i\mathbf g\mathbf h\mathbf t\mathbf s=1/\mathbf v\mathbf i.\mathbf c,\mathbf d\mathbf a\mathbf t\mathbf a=\mathbf d\mathbf a\mathbf t,\mathbf m\mathbf e\mathbf t\mathbf h\mathbf o\mathbf d=\text{"}\mathbf{HS}\text{"})\\\mathbf r\mathbf e\mathbf s\end{array}$$

**Fig. 6:**
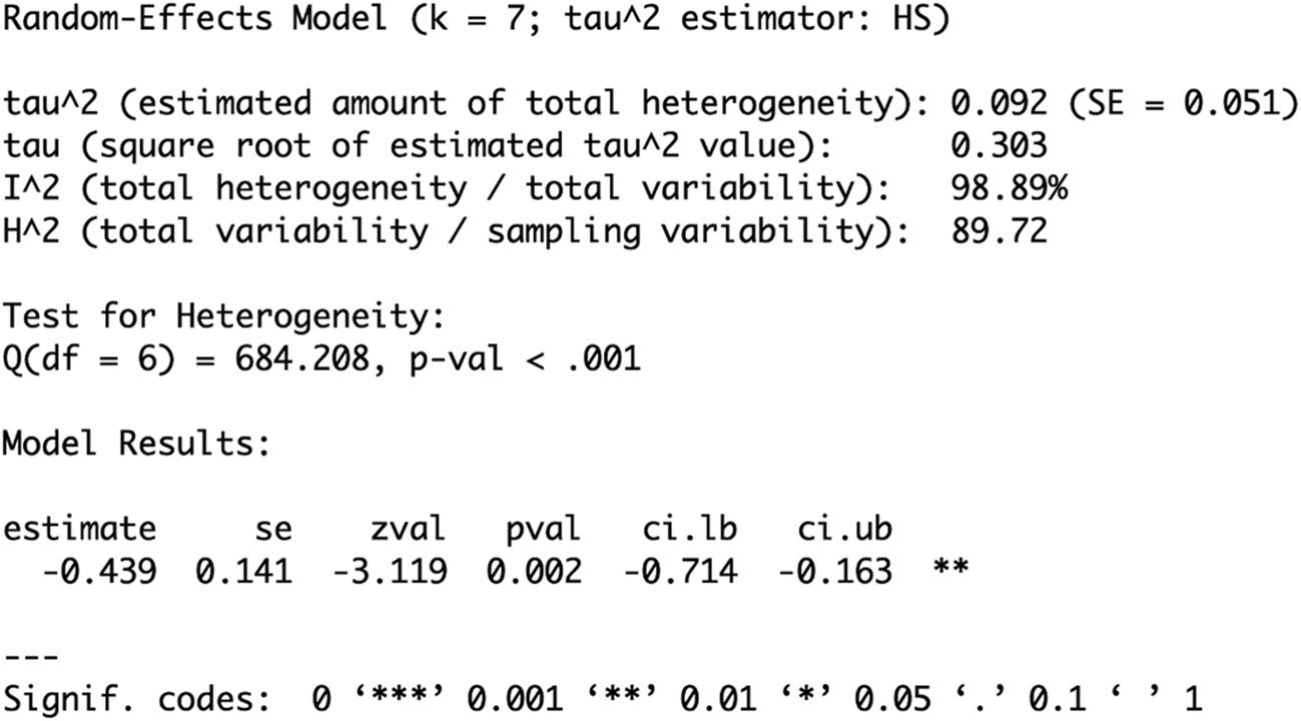
Output of your Psychometric Meta-Analysis

You will arrive at the following output (see Fig. 6):

As you see from the I^2^ value, the heterogeneity is reduced by the suggested reliability correction. This example illustrates that heterogeneity is partially explained by artifacts and not completely by moderator variables through psychometric corrections.

The same analysis example should be repeated if the study database consists of online and offline data collection in subgroup analyses (Borenstein et al., 2021; Schmidt & Hunter, 2014). In that case, each study sample, either online or offline, should be used for separate meta-analyses and the aggregated Odds Ratio values of the two meta-analyses should be compared.

If any individual data are available, it is also recommended to use these individual data as the unit of analysis instead of the aggregated study unit to prevent any aggregation bias. The same R template above can be used for these analyses.

#### Summary

We outlined the primer for meta-analyses on Internet-based research data and introduced the advantages of Internet-based research for conducting an ideal meta-analysis (e.g., easy storage of data, data collection process comparison). We also guided the interested reader through a practical analysis example for this type of meta-analysis that will be most common in the future.

We will now provide an overview of Internet-based research that gives an overall picture of the field’s research status and the quality of the meta-analyses conducted. Furthermore, we would like to answer the question whether meta-analyses on Internet-based research meet the optimal precondition for IPD meta-analyses and use their data potential optimally. The overall aims of the following overview are: to summarize the meta-analyses in the field of Internet-based research and to identify research gaps in order to provide researchers with a starting point for future studies and/or meta-analyses.

## Overview

### Research questions

What is the current state of meta-analyses in the field of Internet-based research, considering their methodological approaches and their scope? The overall goal is to provide a map of meta-analyses in the field of Internet-based research while examining the methodological practices of these meta-analyses. Therefore, we are interested in the following:

1. What are the best practices for meta-analyses in the field of Internet-based research regarding (a) the type of meta-analysis (e.g., IPD meta-analysis) and (b) the comparison of different data types (online vs. offline)?
2. What is the scope of these meta-analyses? Are there any research gaps or missing topics that need to be addressed in future research, or are there already several meta-analyses providing a rich variety of evidence-based recommendations for the field of Internet-based research?

### Method

**Fig. 7:**
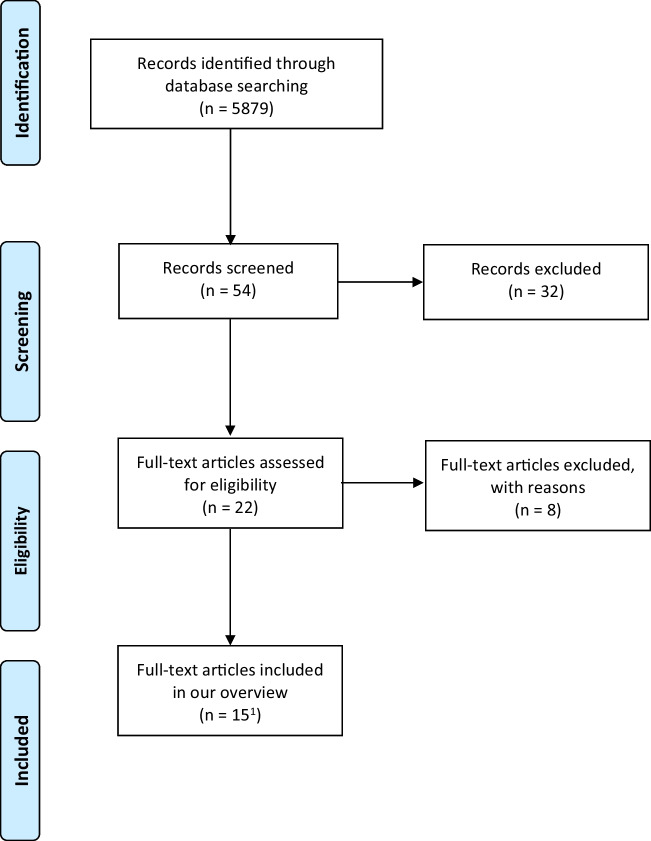
The Process of Identifying the 15 Meta-Analyses for Our Overview on Internet-Based Research. Adapted PRISMA Flow Chart for Overviews by the Authors

^1^thanks to a reviewer recommendation after our literature search an additional article was added (Edwards et al., 2009).

To answer the research questions, we conducted a comprehensive literature search for meta-analyses on Internet-based research. We then selected meta-analyses for the overview based on a set of criteria and coded them accordingly. In the following sections, we describe each step taken to reach our final database in detail. We present an overview of our literature search in Figure 7, the PRISMA flow diagram (Moher et al., 2009). This figure shows the search, retrieval, and coding processes of the meta-analyses. As the PRISMA flow diagram was developed for meta-analyses, we adapted it for our overview.

#### Literature search

**Fig. 8:**
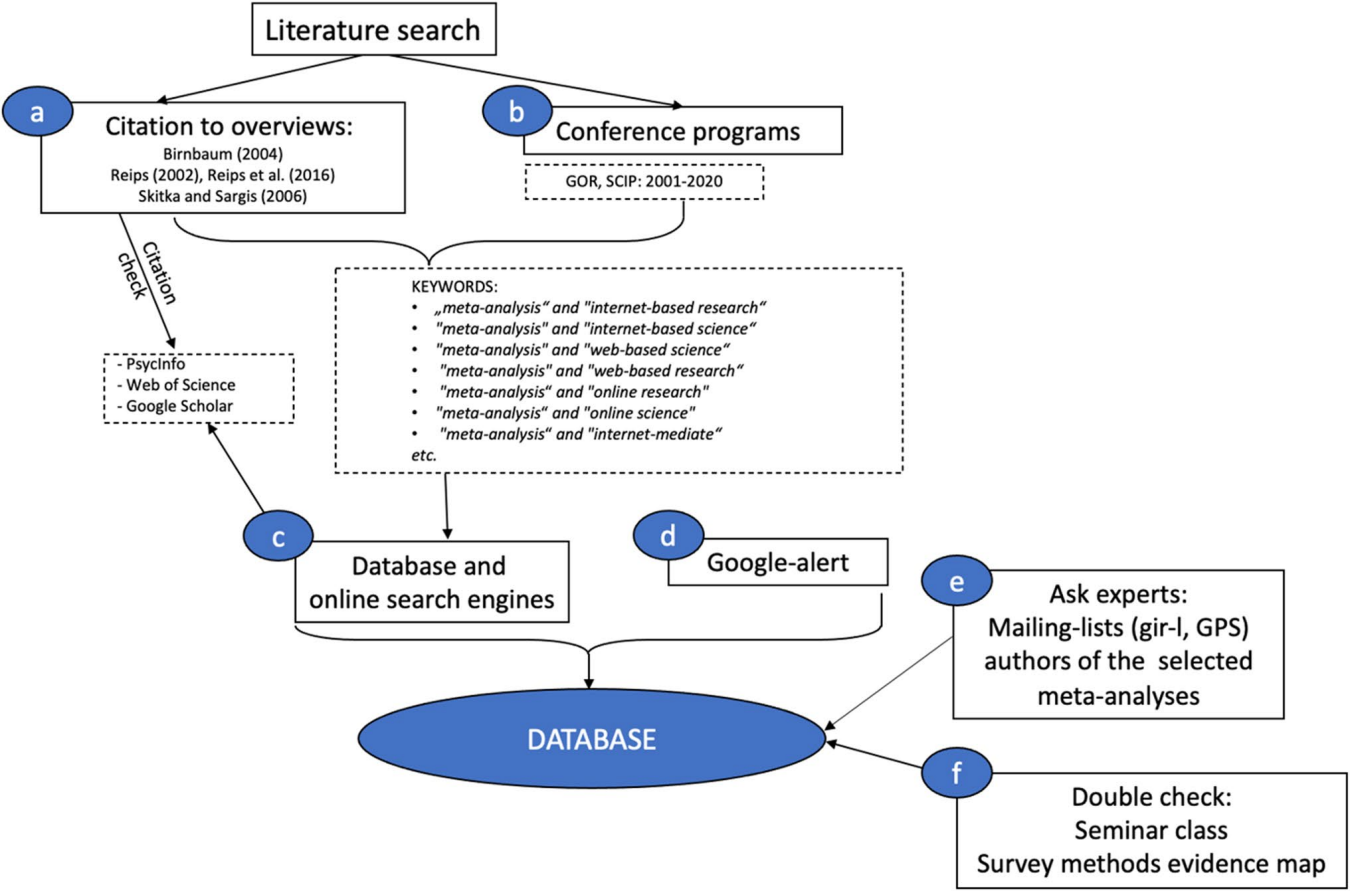
The Four Different Search Strategies (a–d) and Two Control Strategies (e, f) Used in Our Search for Meta-Analyses

To identify relevant meta-analyses for our overview, six different strategies were applied (see Figure 8). The starting point for the overview were key publications in the field of Internet-based research (Birnbaum, 2004; Reips, 2002, 2021; Reips et al., 2016; Skitka & Sargis, 2006).

Our literature search consisted of four search strategies. We obtained relevant keywords from (a) previous articles and (b) conference programs. Then we (c) used these keywords in our search in different databases and (d) created a Google alert for the keywords so that we would be notified of recently published articles. We also used two control strategies to check our database. We (e) asked experts (e.g., authors of the meta-analyses) to double-check the database and asked researchers in the field if they knew of or planned any meta-analyses. Moreover, we (f) double-checked our sample of meta-analyses against the sample of meta-analyses in the survey research evidence gap map by the GESIS-Leibniz Institute for the Social Sciences.^2^

We provide a detailed description of our literature search in the Appendix (see Literature Search and Table 2). We conducted the literature search in March 2021 and updated it to the best of our ability (see Fig. 7 for the identification and screening).

#### Eligibility criteria

For our overview of meta-analyses in the field of Internet-based research, we applied the following three inclusion criteria:

1. Time: Although our search was not restricted to a time window, we found no meta-analyses that were conducted before 2000.
2. Language: We searched for studies published in English or German.
3. Type of study: We considered reviews that focused on a quantitative evaluation of their study sample (e.g., Porter et al., 2019; Sheehan, 2006). Therefore, the overview had to be a meta-analytic summary.

We also excluded meta-analyses that had replicas and were thus outdated (e.g., original version: Manfreda et al., 2008, and current version: Daikeler et al., 2020) as well as meta-analyses that did not fit the methodological scope required for our overview (e.g., meta-analyses on mode effects for specific tests, meta-analyses on social anxiety and Internet use, etc.; for details, see Table 3 in the Appendix).

#### Coding studies

The final database for coding consisted of 15 articles containing 24 meta-analyses (see Figure 8, procedure eligibility included). We coded each of these meta-analyses in relation to publication and meta-analysis features (for details, see coding sheet in Table 4 in the Appendix).

### Results

First, we report the features related to our research questions, namely, the types of meta-analyses and data comparison, followed by the meta-analyses’ scopes (see Table 1). We summarize the presented literature and coding and meta-analyses’ features in Tables 5 and 6 in the Appendix.

**Table 1:** Publication and Meta-analysis Features of Our Sample (N = 24)

Authors	Number of participants	Time range	Number of studies	Number of effect sizes Psychometric IPD			Data comparison	PB conducted?	PB results	Outlier Analysis	Scope
Ferrer et al. (2015)	not mentioned (1)	2004-2014	26	89	no	X	no	yes	mixed results	no	Affect induction
Daikeler et al. (2020)	not mentioned	2005-2016	82	114	no	X	multiple	yes	no	yes	Response rate
Weigold et al. (2019)	55921	1992-2013	73	96	no (3)	X	yes	yes	no	yes	
Villar et al. (2013)	not mentioned	2001-2011	10	32	no	X	no	no	no	yes	
Medway & Fulton (2012)	17547	2001-2011	16	16	no	X	yes	yes	no	no	
Edwards et al. (2009)	about 2000	1940-2008	158	513	no	X	yes	yes	mixed results	no	
Shih & Fan (2009)	53158	1992-2006	29	35	no	X	yes	yes	no	yes	
Shih & Fan (2008)	2616	1998-2006	37	39	no	X	yes	no	no	no	
Shih & Fan (2007, 1)	463563	1996-2006	35	43	no	X	yes	no	no	yes	
Shih & Fan (2007, 2)	471407	1996-2006	40	52	no	X	yes	no	no	yes	
Cook et al. (2000)	not mentioned	1994-2000	39	56	no	X	no	no	no	no	
Göritz (2006, 1)	212810	1999-2005	10	32	no	X	no	yes	no	yes	Incentive
Göritz (2006, 2)	7073	1999-2005	9	26	no	X	no	yes	no	yes	
Gnambs & Kaspar (2017, 1)	3746	2001-2013	12	30	no	X	yes	yes	no	yes	Socially desirable responding
Gnambs & Kaspar (2017, 2)	2951	2003-2014	9	66	no	X	yes	yes	no	yes	
Gnambs & Kaspar (2017, 3)	16034	2000-2014	28	96	no	X	yes	yes	no	yes	
Doudou & de Winter (2014)	16750	1969-2014 (2)	51	63	no	X	yes	yes	no	no	
Callegaro et al. (2015, 1)	not mentioned	1994-2012	17	17	no	X	multiple	no	no	yes	Item-Format
Callegaro et al. (2015, 2)	not mentioned	1994-2012	17	17	no	X	multiple	no	no	yes	
Callegaro et al. (2015, 3)	not mentioned	1994-2012	17	17	no	X	multiple	no	no	yes	
Callegaro et al. (2015, 4)	not mentioned	1994-2012	17	17	no	X	multiple	no	no	yes	
Callegaro et al. (2015, 5)	not mentioned	1994-2012	17	17	no	X	multiple	no	no	yes	
Callegaro et al. (2015, 6)	not mentioned	1994-2012	17	17	no	X	multiple	no	no	yes	
Cornesse & Bosnjak (2018)	not mentioned	1989-2016	66	101	no	X	multiple	no	no	yes	Representativness

(1) They only mentioned participant age information; (2) The large time range is linked to the comparison of Internet connectivity with offline mode (computers linked in a local network were also coded as offline); (3) Corrections, but not psychometric corrections.

#### Meta-analyses’ features

##### Types of meta-analyses

There were no IPD or psychometric meta-analyses on Internet-based research (see Table 1). As we outlined before, due to technological advances, IPD meta-analyses could be easily conducted in Internet-based research. However, there were no psychometric meta-analyses conducted on the study-aggregation level. Therefore, in our view, the potential of Internet-based data gathering has not been fully exploited in meta-analyses. *Online and Offline Data Comparison*. We argue that online versus offline differences in meta-analysis data collection should be considered. Nearly a quarter of the 24 meta-analyses did not consider any mode differences because they were restricted to one mode (five meta-analyses from four articles: Cook et al., 2000; Ferrer et al., 2015; Göritz, 2006; Villar et al., 2013). Ferrer et al. (2015) compared the online versus offline modes indirectly by comparing their meta-analysis results with another meta-analysis on offline data-gathering approaches. Additionally, Villar et al. (2013) studied the differences in progress indicators only for web surveys (mono-mode).

For the most part, the meta-analyses in our overview compared at least two different modes of data gathering. Callegaro et al. (2015) described the different modes in detail but did not include any mode differences in their analyses. Although they did not consider any data collection differences^3^, we have categorized their analysis as using a multi-mode approach.

#### Scope of meta-analyses

The scopes of the meta-analyses we considered, operationalized as the dependent variables, were mostly the *response rate* (10 out of 23; 43%, nine different articles), followed by *data quality based on socially desirable responding* (4 out of 23, 17%, only two articles), and *item format check* (6 out of 23, 26%, only one article; see Table 1 for additional scopes). While six meta-analyses were conducted on item format, they were published within a single article (Callegaro et al., 2015), implying that the attention given to this topic may be overestimated if we focus only on the number of meta-analyses conducted.

Only one article was published on the methodological influence of *incentives*^4^ (Göritz, 2006). She reported two meta-analyses (two out of 23, 8%)—one on participant response (i.e., the number of participants who call up the first study page) and one on retention (the number of responding participants who reach the last page of the study). Finally, only one meta-analysis each focused on *affective induction in Internet-based studies* (Ferrer et al., 2015, one out of 23, 4%) and *representativeness* (Cornesse & Bosnjak, 2018, one out of 23, 4%).

Due to the different scopes of the meta-analyses, we present the study outcomes according to the main group of dependent variables: *response rate* versus *others* (data quality and answering behavior as well as representativeness).

##### Response rate

Most of the studies used the response rate as a dependent variable. The response rate is crucial in survey research because a low response rate has the potential to introduce non-response bias. Non-response bias may cause the survey results to be misleading.

Daikeler et al. (2020), in their comparison of web surveys and other modes with regard to the response rate, showed that web surveys have a lower response rate compared to other modes, implying that differences exist between online and offline data gathering. Several other meta-analyses were in line with this result as well. Medway and Fulton (2012) showed in their meta-analysis that mail surveys that incorporate a concurrent web option have significantly lower response rates than those that do not. In all meta-analyses by Shih and Fan, mail surveys had a higher response rate compared to both e-mail surveys (Shih & Fan, 2009) and web surveys (Shih & Fan, 2007, 2008). Furthermore, Weigold et al. (2019) found a higher response rate for paper-and-pencil surveys compared to computer-based surveys.

Cook et al. (2000) focused only on online surveys and showed that the number of contacts, personalized contacts, and pre-contacts increased the response rate. Edwards et al. (2009) conducted several meta-analyses on the effect of methods to increase response rate to postal and Internet-based surveys. For Internet-based surveys, they found that certain types of survey context (incentive, origin, communication) or survey characteristics (length, appearance, context, origin) had a positive impact on the response rate.

Moreover, the meta-analysis by Villar et al. (2013) that we introduced as a template in the primer above was only on web surveys; they found that, in progress indicators for web surveys, the speed had to be considered. In the best-case scenario, fast-to-slow indicators reduced the drop-out rate and slow-to-fast indicators increased drop-out rates. They should both be avoided as these techniques are misleading the respondents.

To summarize, in terms of the response rate, the aforementioned meta-analyses primarily compared online versus offline modes of data and revealed the differences between them. Furthermore, the meta-analysis by Göritz (2006) indicated that providing incentives was more likely to prompt participants to begin a web survey and stay with it, as compared to when no incentive was provided.

In the following section, we report on the answering behavior and data quality in Internet-based research and relate it to representativeness.

##### Data quality, answering behavior and representativeness

The other half of meta-analyses, 11 of them, covered the topic of data quality and answering behavior.

Two articles (Dodou & de Winter, 2014; Gnambs & Kaspar, 2017) on socially desirable responding showed no differences between paper-and-pencil and web-based or computerized surveys. Notably, the meta-analysis on social desirability in Internet connectivity (Dodou & de Winter, 2014) traced back to studies on computers without Internet connectivity. Hence, Internet connectivity means access to the Internet vs. computers without any Internet access (separate computer). Due to different testing situations (Reips, 2002), future studies should separate *computer versus Internet connectivity* and compare the differences.

The only meta-analysis on *answering formats* compared forced-choice with check-all items. Callegaro et al. (2015) showed a higher endorsement rate and a longer response time in the forced-choice format than in the check-all format. According to the meta-analysis by Ferrer et al. (2015), affect can be effectively induced in Internet-based studies, with the exception of happiness.

The meta-analysis by Cornesse and Bosnjak (2018) focused on survey characteristics and representativeness and showed that web surveys were less representative than single-mode surveys. However, they acknowledged the limitation that their results were based only on a single representative measure (median absolute bias [MAB]) and ignored additional measures (R-indicators; for details, see Cornesse & Bosnjak, 2018).

To summarize, there are few meta-analyses on data quality in Internet-based research, and these meta-analyses are quite heterogeneous. Considering the relevance of Internet-based research, the lack of more meta-analyses on data quality is surprising.

## Discussion

Since the development of meta-analysis research in the last century, the impact of Internet-based research has steadily increased in recent years, which has led to meta-analytic research syntheses. In our primer, we show first the need to consider the specifics of Internet-based research for meta-analysis research to optimally address and utilize data from Internet-based research. Our follow-up overview on Internet-based research shows that 24 meta-analyses have been published in 15 articles within the field. While Internet-based research has existed for over 25 years, only a few meta-analyses have been published. In line with our primer recommendations on meta-analyses on Internet-based research, our overview shows that there are mostly online and offline data-collection comparisons, but contrary to our recommendation, no IPD or psychometric meta-analyses have been conducted in the field of Internet-based research. Therefore, the potential of Internet-based research, especially for IPD meta-analysis (i.e., preventing any aggregation bias), has been neglected so far.

Our overview reveals an *information gap for individual participant data*. In addition, it shows that participant information, such as their number, their age, or their nationality, is rarely reported in meta-analyses. We see the need to report more individual data (i.e., at least the gender and age of the participants included in the original studies) for conducting comprehensive IPD meta-analyses. However, this problem is not limited to the field of Internet-based research; rather, it is found in other fields, such as education, as well (Südkamp et al., 2012).

Furthermore, our overview shows a clear need for psychometric meta-analyses within the field, as the impact of study artifacts (e.g., measurement error) is evident in the field of Internet-based research. If only bare-bones meta-analyses are conducted, then there is a danger of overestimating the data heterogeneity and maybe wrongly introducing moderator variables.

Our examination of robustness analyses, such as publication bias estimation and outlier analysis, shows that most of the recent meta-analyses follow these recommendations. Moreover, robustness may be underestimated, as some authors might have conducted such analyses without reporting them.

As publication bias was present in only a few meta-analyses in our overview, it seems that it is currently not a problem in meta-analyses of Internet-based studies. This may be because the field is new and the review process in the field is conducted in fair ways, thus not leaving any study unpublished due to its results. However, as publication bias estimations are still in the development stage and more complex approaches are needed (see, e.g., Dickersin, 2005; Fernández-Castilla et al., 2021), current methods are criticized, and new methods may uncover publication bias in the future.

Taken together, the quality of meta-analyses in the field of Internet-based research is reasonable, though they have yet to fulfill their potential. An online data-gathering approach has the potential to store individual data easily for further evaluation. It must be noted that the methodology of meta-analyses is in continuous development. Newer meta-analyses are not as static as the ones considered in our overview. Static meta-analyses are limited in terms of their database and they soon become outdated, if new studies are published. On average, meta-analyses are out of date after 5.5 years (Shojania et al., 2007) and for those on Internet-based research they may actually age even faster, as data collection technology and methodology is evolving at high speed (Reips, 2021). Conducting a meta-analysis is time-consuming, especially if our suggested best-practice approach is applied: a meta-analysis based on IPD. Such meta-analyses should also be replicated with new statistical aggregation approaches.

We expect a change in the near future regarding the reporting of research syntheses due to living meta-analysis approaches. As living meta-analyses are updated frequently and include all meta-analyses within a field, potential publication bias may also have less of an impact.

## Overviews

Overviews also help identify *research gaps*. Hence, in addition to the methodological quality of the conducted meta-analyses within the field of Internet-based research, the scopes of the existing meta-analyses are considered. The scopes are response rate, incentives, data quality, answering behavior, and representativeness; however, we can conclude that the response rate, different mode effects’ comparisons, and data quality were the main topics considered in those meta-analyses.

In particular, meta-analyses on response rate seem to be the focus. Interestingly, their development is also noteworthy, as it seems that Edwards et al. (2009) contributions initiated further field-specific meta-analyses, such as within education (see e.g., Wu et al., 2022). Although the response rate in these meta-analyses is different (Burgard et al., 2020; Wu et al., 2022), it seems that there is high heterogeneity within these meta-analyses, which may imply that other factors play a role. We do not believe that this high heterogeneity is completely explained by the mentioned between-studies artifacts (e.g., measurement errors), but it may be worthwhile to check in future meta-analyses the impact of them on response rate in Internet-based and traditional studies across and within fields as we have described in our primer.

Although further information about response rate of Internet-based research is desirable and hence, should be considered in each meta-analysis, we argue that Internet-based research has the potential for additional meta-analytic topics. Accordingly, we recommend more meta-analyses on data quality. The rich variety of recommendations for Internet-based research generated several meta-analysis topics (e.g., methods and techniques; Reips, 2000, 2021; Reips et al., 2016). For example, empirical research following the recommendation to use a high-hurdle technique in Internet-based research yielded contradictory results and interpretations (see, e.g., Göritz & Stieger, 2008, vs. Reips, 2000). To clarify under which condition this technique works, further studies and meta-analyses on this topic are highly recommended.

Another issue not covered by meta-analyses on Internet-based research is *late responding*; neglecting this subgroup of responding participants may also impact the generalizability of Internet-based research. However, the following elements are not yet known: how much effort is worthwhile when reaching out to late responders, how large is the share of late respondents in web-based surveys compared to mail surveys, and whether there are any differences between these proportions (see Laupper et al., 2023).

Finally, while several of the reviewed meta-analyses focused on the response rate, future studies or meta-analyses should focus on the recruitment of participants to answer, for instance, the question of whether the “multiple site entry technique” delivers the promised results. Reips (2000, 2002) suggested linking an online survey (or experiment) to different sites; as a result of this strategy, the different access points to the Internet-based study may reveal the different effects the different samples have when comparing their results. In his recent review of web-based research in psychology, Reips (2021) summarized several articles that used the multiple site entry technique.

Besides the advice for upcoming meta-analytic topics, we highlight that our overview provides an ideal basis to extend the PRISMA guidelines. Currently, there are different guidelines under development for specified study types, e.g., with children (PRISMA-Child). Hence, our overview provides an ideal basis for the development of PRISMA for studies conducted on Internet-based research in the future, making researchers aware of the additional data and analysis potential of Internet-based research compared to traditional studies.

Taken together, our overview shows that several meta-analyses have been conducted in Internet-based research and that their quality is in line with the current state-of-the-art practices in meta-analysis research. Future meta-analyses should focus on data quality and answering behavior and also consider the potential of Internet-based research, namely for easily collecting, storing, and using individual-based data for more complex IPD meta-analyses in the future; this will also facilitate an update of our overview.

### Electronic supplementary material

Below is the link to the electronic supplementary material.

Supplementary file1 (TXT 5 KB)

Supplementary file2 (TXT 0 KB)

Supplementary file3 (TXT 0 KB)

## Appendix

### Literature search

We checked the citations of these key publications in each search engine or database. We used the keywords we extracted from these key publications for the literature search. To include both U.K. and U.S. articles, we considered both English spellings, which sometimes differ slightly (e.g., meta-analyse vs. meta-analyze). Our comprehensive keyword list is displayed in Table 2 in the Appendix. For our search, we used the following search engines and databases: *APA PsycInfo* (active since 1906, international literature on psychology) and *Web of Science* (journals on humanities, social sciences, and natural sciences). In addition, we used the online search engine *Google Scholar*.^5^ Afterward, we scrutinized the selected literature for relevant keywords in the title and the abstract. We also checked the reference list for relevant cited literature.

To also collect grey literature, we checked related conference programs, such as the General Online Research (GOR) Conference 2021^6^ and Society for Computation in Psychology (SCIP from 2001 to 2020^7^), for relevant literature.

Finally, we e-mailed researchers in the field of Internet-based research (GOR-mailing list and German Psychological Society) to ask if they were preparing a meta-analysis at the time; this strategy prevented us from missing any meta-analysis in preparation. We double-checked our literature search with several databases. Similarly, we contacted the first authors of each of the included meta-analyses and asked if they were currently working on or planning on conducting any additional meta-analyses. With the exception of Medway and Fulton (2012), we had the contact information for at least one author of each article. Four of the contacted authors answered within one month. From two authors, we received additional pointers on meta-analyses.

We also created different Google alerts with the keywords to ensure we would be informed about ongoing work on this topic, such as newly launched projects or publications. A seminar class also double-checked our literature search as a training exercise. Finally, we double-checked the selected meta-analyses against the survey research evidence gap map by the GESIS-Leibniz Institute for the Social Sciences.

see Tables 1, 2, 3, 4, 5 and 6

**Table 1:** Recommend databases

Search engines	Access link
Web of Science	https://www.webofknowledge.com/
PsycInfo	https://www.apa.org/pubs/databases/psycinfo
Google scholar	https://scholar.google.com

**Table 2:** List of Key Terms Used for Our Literature Search. Overall, the key terms used were a combination of terms representing (a) Internet-based research and (b) meta-analysis. Additionally, we used specific terms (c) representing recommendations for conducting Internet-based research. We considered U.K. as well as U.S. English terms (e.g., meta-analyse and meta-analyze)

Key terms:
a)
• computer
• computers
• internet
• internet-based research
• internet-based science
• internet-mediate
• online research
• online science
•www
• web-based research
• web-based science
b)
• meta-analysis
• meta-analyses
• review
• research synthesis
• systematic review
c)
• high hurdle technique
• multiple site entry technique
• seriousness check
• warm up technique

**Table 3:** Additional Reasons for the Exclusion of Meta-Analyses or Studies

a. If there was a replication or an extension of a previous meta-analysis, we only considered the most recent version (e.g., original version: Manfreda et al., 2008, and current version: Daikeler et al., 2020).
b. Studies without a clear separation between Internet-based and computer-based methods were not considered. One example is the study by Steger et al. (2020); the focus of their study was proctored versus unproctored ability assessments, and Internet-based and computer-based studies were mixed up. However, it should be noted that, in their available database, there is only a small subsample of proctored ability assessments, which is not within the scope of our review.
c. We excluded studies comparing specific tests on computerized and survey versions (e.g., Finger & Ones, 1999).
d. Studies on social anxiety and Internet use were also excluded (Prizant-Passal et al., 2016).
e. We excluded meta-analyses that checked any mode effect for specific tests (e.g., reading for K-12; see Wang et al., 2008).
f. We excluded, for instance, the study by Griffiths et al. (2006) because the applied field, rather than methodological issues, was the focus of their review.

**Table 4:** Coding Sheet Applied to the Considered Meta-Analyses Sorted by the Publication and Meta-Analysis Features

**1)** Publication	
Publication year	The publication year of the meta-analysis (e.g., 2001)
Publication outlet	The type of publication outlet either as a journal (= 1), conference contribution (= 2), or PhD thesis (= 3)
Authorship	Name of the author(s) (e.g., Reips)
**2)** Meta-analysis	
Literature search	
Unpublished literature	If unpublished literature is considered or only published
Search engines	If search engines are listed
Keywords	If the used keywords for the literatures search are reported
PRISMA	If a PRISMA flow chart is presented.
Coding procedure	
Codes	If the applied codes are reported and defined
Coders	If the detailed number of coders is reported
Agreement	If the consensus-finding procedure is described
Database	
Number of participants	Number of participants considered in the meta-analysis (e.g., 111)
Participants’ characteristics	Age (years), gender (% female), education (= Highschool or not)
Time range	Time range of the considered original studies within the meta-analysis (e.g., 2002–2015)
Number of studies	Number of studies considered in the meta-analysis (e.g., 34)
Number of effect sizes	Number of effect sizes considered in the meta-analysis (e.g., 45)
Type of meta-analyses	
Psychometric	If the meta-analysis used a psychometric meta-analysis approach according to Schmidt und Hunter (2014) (yes = 1, no = 2)
IPD	The aggregation unit of the meta-analysis: If the meta-analysis used individual participant data and ran an individual participant data meta-analysis (yes = 1, no = 2)
Type of analyses	
Data comparison	If the meta-analysis used different type of data and compared them, meaning offline and online data comparison (yes = 1, no = 2)
PB conducted?	If a publication bias estimation was conducted when running the meta-analysis (yes = 1, no = 2)
PB results	If, after running a publication bias estimation, a publication bias was indicated (yes = 1, no = 2)
Outliers	If an outlier analysis was considered when conducting the meta-analysis (yes = 1, no = 2)
Scope	
Results	The reported meta-analysis outcome (e.g., incentive has an impact)
Topic	The meta-analysis content (e.g., social desirability responding)

PB = Publication bias.

### Note About the Literature Search and Coding Procedure

#### Literature search

We checked whether the authors of our sample of meta-analyses also considered unpublished literature to prevent any publication bias. We also examined whether they listed the search engines and keywords used. Finally, we checked whether the meta-analyses used any type of PRISMA flow chart.

#### Coding procedure

To assess the quality of the coding procedure used in the meta-analyses, we examined several features of the coding procedure. We checked whether the authors reported the different codes they used and how different coders reached a consensus—for instance, whether they reported any agreement reliability or not.

**Table 5:** Features of the Literature Research Reported in Our Sample of Meta-Analyses (Ordered According to the Publication Year)

Article	Unpublished literature	Search engines	Keywords	PRISMA
Daikeler et al. (2020)	Yes	Yes	Yes	Yes
Weigold et al. (2019)	Yes	Yes	Yes	Yes
Gnambs & Kaspar (2017)	Yes	Yes	Yes	Yes (partly)
Callegaro et al. (2015)	Yes	Yes	Yes	No
Ferrer et al. (2015)	Yes	Yes	Yes	Yes
Doudou & de Winter (2014)	Yes	Yes	Yes	Yes
Villar et al. (2013)	Yes	Yes	Yes	No
Medway & Fulton (2012)	Yes	Yes	Yes	No
Edwards et al. (2009)	Yes	Yes	Yes	No
Shih & Fan (2009)	Yes	Yes	Yes	Yes (partly)
Shih & Fan (2007)	Yes	Yes	Yes	No
Göritz (2006)	Yes	Yes	Yes	No
Cook et al. (2000)	Yes	Yes	Yes	No

All meta-analyses except one (Shih & Fan, 2008) also considered unpublished literature. All meta-analyses provided information about the search engines and keywords used. Not all meta-analyses considered reporting their literature search by a PRISMA flow chart. However, when considering only recent meta-analyses, it can be seen that recent meta-analyses in the field on Internet-based research have reported their literature search by a PRISMA flow chart. Overall, the literature search in our sample of 24 meta-analyses followed the guidelines on meta-analysis research.

**Table 6:** Features of the Coding Procedure Reported in Our Sample of Meta-Analyses (Ordered According to the Publication Year)

Authors	Codes	Number of Coders	Agreement
Daikeler et al. (2020)	yes	yes	Krippendorff's Alpha (0.92)
Weigold et al. (2019)	yes	no	no
Cornesse & Bosnjak (2018)	yes	yes	consensus
Gnambs & Kaspar (2017)	yes	2 (subsample)	Cohen's Kappa: .99 -1.00, consensus
Callegaro et al. (2015)	no	no	no
Ferrer et al. (2015)	yes	2 (subsample)	ks .48, consensus
Doudou & de Winter (2014)	yes	no	no
Villar et al. (2013)	yes	no	no
Medway & Fulton (2012)	yes	2	consensus
Edwards et al. (2009)	yes	2	Kappa, consensus
Shih & Fan (2009)	yes	no	no
Shih & Fan (2008)	yes	no	no
Shih & Fan (2007)	yes	no	no
Göritz (2006)	yes	no	no
Cook et al. (2000)	yes	yes	consensus

The table shows that, for the most part, codes are presented. However, the number of coders is seldom reported, and agreement measures (e.g., Krippendorff's alpha) are rarely calculated. Hence, compared to the description of the literature search procedure, the coding procedure is reported in less detail.

## Footnotes

1 For a detailed overview of the impact of research synthesis for practice, see Wyborn et al. (2018).

2 https://egmopenaccess.3ieimpact.org/evidence-maps/gesis-survey-methods-evidence-map

3 Interestingly, the authors concluded that effects are remarkably consistent across modes (paper and pencil or web); see Callegaro et al. (2015, p. 219).

4 We highlight that Edwards et al. (2009) considered mode effects on responses and considered different types of incentives. Because their focus was the response rate this study was classified under the topic responses rate.

## Declarations

### Open practices statement

Because our overview does report already published data, no original data for the overview are available. This overview is not preregistered. However, the papers considered in our overview are available on request for individual, non-commercial use; esther.kaufmann@uni-konstanz.de.
